# COST ANALYSIS OF MOTORCYCLE ACCIDENT VICTIMS AT A UNIVERSITY HOSPITAL: PERSPECTIVES FROM 2017 AND 2020

**DOI:** 10.1590/1413-785220233101e258318

**Published:** 2023-04-17

**Authors:** Amanda Baptistella, Henrique Carvalho e Silva Figueiredo, Carlos Augusto de Mattos, Cintia Kelly Bittar

**Affiliations:** 1 Pontifícia Universidade Católica de Campinas, Faculdade de Ciências Médicas, Campinas, SP, Brazil.; 2 Hospital PUC-Campinas, Campinas, SP, Brazil.

**Keywords:** Accidents, Traffic, Hospitalization, Costs and Cost Analysis, Acidentes de trânsito, Hospitalização, Custos e Análise de Custo

## Abstract

**Introduction:**

Motorcycle accidents constitute a public health problem that affects public and private health services due to the expenses of the victim's treatment and rehabilitation.

**Objective:**

Evaluate the impact of motorcycle accident costs in a university hospital in 2020.

**Method:**

Comparative analysis of the costs of motorcycle accident patients in 2020 and 2017.

**Results:**

Among 151 patients included in the study, the average cost was U$3,083.54, and the average days of hospitalization were 5.3 days. The patient with the highest cost to the hospital spent U$22,504.05, and the patient with the lowest cost spent U$356.72. The longest stay among these patients was 41 days, and the shortest was one day. The average cost per patient per day for the entire sample was U$581.80.

**Conclusion:**

The formulation and application of strategies that promote the reduction of motorcycle accidents in the city of Campinas are necessary. ***Level of evidence II, Retrospective study.***

## RESUMO

**Introdução:**

Acidentes motociclísticos constituem um problema de saúde pública que atinge os serviços públicos e privados de saúde, em função dos gastos com o tratamento e com o processo de reabilitação da vítima.

**Objetivo:**

Avaliar o impacto dos custos dos acidentes motociclísticos em um hospital universitário em 2020.

**Método:**

Análise comparativa dos custos dos pacientes vítimas de acidente motociclístico no ano de 2020 e 2017.

**Resultados:**

Dentre 151 pacientes incluídos no estudo, o custo médio foi de U$3.083,54 e a média de dias de internação foi de 5,3 dias. O paciente que apresentou maior custo para o hospital, teve um gasto de U$22.504,05 e o que teve o menor custo, gastou U$356,72. O maior tempo de internação, entre estes pacientes, foi de 41 dias e o menor tempo foi de 1 dia. O custo médio por paciente por dia, em toda a amostra, foi de U$581,80.

**Conclusão:**

Faz-se necessário a formulação e aplicação de estratégias que promovam a redução dos acidentes motociclísticos na cidade de Campinas. ***Nível de evidência II; Estudo retrospectivo.***

## INTRODUCTION

Traffic accidents have had an important impact on the health of populations in several countries around the world. According to the World Health Organization, around 1.24 million people die each year due to traffic accidents, and it is the leading cause of death among young people aged 15 to 29 years.^1,2^

Brazil, in recent decades, has gradually been ranked among the world champions in traffic accidents, with emphasis on accidents involving motorcycles. This vehicle has increasingly gained acceptance and approval from the population.^3^

The COVID-19 pandemic had dramatic consequences for the health system organization. Although orthopedics and traumatology, as medical specialties, do not deal directly with the effects caused by SARS-CoV-2, its performance was significantly affected, especially at Hospital PUC-Campinas, due to its qualification as a reference for the care of traumas and “non-covid” pathologies.^4,5^

The study of direct cost in health aims to quantify, in monetary values, how much resources were used directly in the treatment/ intervention of a patient and can be subdivided into medical (fees, hospitalization, medication, etc.) and non-medical (transportation) costs patient, feeding, etc.). Indirect costs refer to the loss of working time of the patient or their families due to the disease or its treatment, which can be measured, for example, in terms of lost productivity.^6^ Therefore, the dimension of costs is quite broad, and only a portion of them was addressed here. Furthermore, the present study analyzed only direct medical expenses related to hospital admissions. The time interval for analysis is restricted to one year, and the examination of data relating to a single university hospital is another limitation.

The present study aimed to describe and compare hospitalization rates for motorcycle accidents at a university hospital in 2020, estimate the direct medical cost and length of stay of hospitalizations for motorcycle accidents.

## METHODS

That is a cross-sectional, descriptive, comparative, and retrospective study, with a quantitative approach, based on the cost reports of patients victims of motorcycle accidents conducted between January/2020 and December/2020, as it aims to observe, record, and analyze the costs of Hospital PUC-Campinas with this group. The Research Ethics Committee filed this study with registration on Plataforma Brasil under number 88812818.30000. We waived the patient consent because we retrospectively analyzed previously collected anonymous data; the Research Ethics Committee approved the use of data for these purposes.

All patients victims of motorcycle accidents hospitalized for those 12 months at the Hospital PUC-Campinas, located in Campinas, covering the entire Northwest region of the city, were included. The institution also received cases from other areas of Campinas because it qualified as a reference to caring for the flow of “noncovid” traumas and pathologies.^5^

We defined a victim of a motorcycle accident upon entering the service at Hospital PUC-Campinas. We identified the patients from the emergency Surgery and Trauma Service team - SCUT of the hospital.

We obtained the information on hospital costs for each patient from our hospital cost accounting database. The cost per patient method was applied, considering the value of the hospital rate, procedures, direct product, third party, fee, and days of hospitalization. We included a comparison of the cost between patients victims of motorcycle accidents in 2020 and patients in 2017, using data collected by the “Socioeconomic impact of motorcycle accident victims in the emergency room of a hospital (Part 2) ”.^7^

Values were adjusted for inflation, understood as when the same nominal amount of money buys less in terms of a fixed basket of goods and services.^8^ Thus, costs were first inflated using the National Consumer Cost Index (IPCA) (Table 1). Values were then converted from the local currency (R$) to U$, using the exchange rate relative to the period during which the cost data was collected (Table 2).

**Table 1:** Basic correction data by IPCA (IBGE).

**Data reported**	
Initial date	01/2017
Final date	12/2020
Final value	R$ 1,00 (REAL)
**Calculated data**	
Correction index in the period	1,16434630
Corresponding percentage value	16,434630 %
Value corrected on the end date	R$ 1,16 (REAL)

**Table 2:** Monthly Average Commercial Dollar Quotation (U$) for Sale in Real (R$).

Months	2017	2020
January	3,197	4,152
February	3,103	4,346
March	3,127	4,894
April	3,140	5,330
May	3,209	5,640
June	3,297	5,203
July	3,205	5,287
August	3,153	5,459
September	3,138	5,403
October	3,196	5,632
November	3,257	5,422
December	3,297	5,142

With this data in hand, we calculated using simple arithmetic the average cost of each patient and the average number of days of hospitalization. According to the trauma mechanism, the average cost of patients and the average number of days of hospitalization for these groups. It was also possible to calculate the average patient/ day cost by comparing the average cost and the average number of days of hospitalization. Categorical variables were presented as absolute frequencies and percentages.

## RESULTS

We included 150 patients during the study period. Table 3 shows the most frequent admissions among patients involved in a collision with automobiles (53.6%).

**Table 3:** Trauma mechanism of patients victims of motorcycles accidents in a university hospital, 2020.

Trauma mechanism	n	%
Motorcycle versus car	81	53,6
Motorcycle crash	40	26,5
Motorcycle versus fixed object	16	10,6
Motorcycle versus motorcycle	6	4,0
Motorcycle versus bicycle	5	3,3
Bike versus animal	3	2,0

Among the 151 patients, the average cost was US$ 3,083.54 (US$ 356.72 - 22,504.05), and the average hospital stay was 5.3 days (1 - 41 days). Thus, the average cost per patient per day across the entire sample was $581.80.

Likewise, compared to 2017, the average cost was U$8,708.77 per patient, and the average hospital stay was 13 (1 - 87 days). The patient with the highest cost had an expense of U$70,689.65 and the lowest, U$972.10. Therefore, the average cost per patient per day was $669.90.

In the present study, for the 81 patients victims of a motorcycle versus car collision (53.6%), there was an average cost of U$ 3,018.27 (U$ 366.74 - 22,488.21). Furthermore, the average length of stay was 5.2 (1 - 41 days), and the average cost was US$580.43 per patient/day.

Among the 40 patients who were victims of motorcycle falls (26.5%), there was an average of 4.5 days of hospitalization (1 - 34 days). The average cost was U$2,786.00 (U$356.72 - 22,196.24), and the average per patient/day was U$619.11.

For the 16 patients victims of a motorcycle versus fixed object collision (10.6%), there was an average cost of U$ 4,482.51 (U$ 948.61 - 14,183.89). The average length of stay was 7.3 (1 - 25 days), and the average cost was U$129.94 per patient/day.

Of the total 14 patients in the sample, victims of less frequent trauma mechanisms (motorcycle versus motorcycle, motorcycle versus bicycle, and motorcycle versus animal), there was an average of 6.3 days of hospitalization (1 - 17). Therefore, the average cost was US$3,250.78 (US$1,049.33 - 7465.2), and the average per patient/ day was US$515.99.

The total value of hospital admissions of patients victims of motorcycle accidents, from January 2020 to December 2020, analyzed in this study, totaled U$ 465,614.82.

## DISCUSSION

The emergency care and traumatology drained a large part of the financial resources of the health sector for the rehabilitation and social inclusion of victims of traffic accidents.^9^ Thus, traffic accidents affect around 1 to 2% of the domestic product (GDP) of low- and middle-income countries, which corresponds to a cost of over 100 billion dollars a year.^10^

In the present study, the 151 admissions for motorcycle-related accidents at the Hospital PUC-Campinas, in 2020 generated a total cost of U$465,614.82 and an average cost of U$3,083.54 per hospitalization in 2020. Differed from that of the average value per admission found in the study “Socioeconomic impact of motorcycle accident victims in the emergency room of a hospital (Part 2)” ^6^ for the same hospital in 2017, with 62 admissions, which was R$ 8,708.77; proving a reduction of 64.5% in costs. Furthermore, there was also a difference concerning the mean length of stay. In the current study, the mean length of stay was 5.5 days, while, in the study mentioned above, it was 13 days.

The increase in the number of hospitalizations is possibly related to the pandemic situation and the expanding market for delivery and motorcycle taxis. According to Diniz et al., the reality of these professionals (motorcycle courier and “motorcycle taxi driver”) surpasses the emotion of riding a motorcycle. Precarious conditions, lack of formal contracts, long hours, stress, psychological pressure for greater productivity, low income, and risks of accidents are constant. The motorcycle courier live with the risk-need dialectic, reported by Veronese & Oliveira: “Between working under the threat of suffering a traffic accident and not working, what to choose? It is up to the motorcycle courier to try to control the risk”.^11^

Furthermore, the central role of Hospital PUC-Campinas in taking care of trauma cases may also be linked to the substantial increase in the number of cases.

Regarding the length of stay, we expected that the incidence of COVID-19 in these patients would increase the span of hospital stay, either by aggravating the patient's clinical condition and prolonging his recovery or by the need to comply with quarantine requirements during the period of hospitalization.^12^ Furthermore, it is plausible that the surgery of patients diagnosed with COVID-19 was postponed, influencing postoperative recovery and possibly contributing to the increase in hospital stay, as shown in other studies.^13-15^

However, as demonstrated above, the reduction in a hospital stay can probably be justified by the recommendations on preparing perioperative environments to support the immediate care of patients with general emergency surgery and traumatized in times of COVID-19 and avoid delays. Moreover, surgical interventions are not limited to patients who are victims of motorcycle accidents.

The “Guia rápido para a atuação dos coordenadores de serviços de Trauma no Brasil”, for example, is general guidelines developed by the Brazilian College of Surgeons (CBC), the Brazilian Society for Integrated Trauma Care (SBAIT), and the Brazilian Chapter of American College of Surgeons, based on those provided by the American College of Surgeons and its Committee on Trauma.^16^ Likewise, these measures and recommendations, together with the reduced length of stay, may justify the reduction in the average cost per patient.

According to the present study, motorcyclists were predominantly victims of a motorcycle versus car collision, representing 53.6%, followed by motorcycle falls (26.5%) and motorcycle versus fixed object collision (10.6%). Thus, the trauma mechanism is essential for predicting injuries, which makes them more suspicious, and, through early diagnosis and treatment, it can often offer the patient a better prognosis.

As a limitation of the present study, it is highlighted that the data refer only to hospitalizations financed by Hospital PUC-Campinas. Therefore, the results presented represent the reality of only one institution.

## CONCLUSION

The data on hospitalization costs arising from accidents involving motorcycles at the Hospital PUC-Campinas, in 2020 only reinforces the need to formulate and apply strategies that promote the reduction of motorcycle accidents in Campinas. We hope that similar studies will provide the basis for adopting prevention policies and improving the care provided to these victims.
